# Scaffold Fixation of Multi-Fragmented Intra-Articular Distal Radius Fractures

**DOI:** 10.1055/a-2811-1965

**Published:** 2026-06-03

**Authors:** Lente H. M. Dankelman, Lucía Chiquiar, Magdalena Hartwich, Mathieu M. E. Wijffels, Alberto Fernandez Dell'Oca, Jesse B. Jupiter, Abhiram R. Bhashyam

**Affiliations:** 1 Division of OrthopaedicsHand and Upper Extremity ServiceMassachusetts General Hospital, Harvard Medical SchoolBostonMassachusettsUnited States; 2 Trauma Research UnitDepartment of Surgery 6993 Erasmus Medical Center: Erasmus MCRotterdamNETHERLANDS; 3 ICUC Medical Learning ProjectMontevideoUruguay; 4 Department of TraumatologyHospital BritanicoMontevideoUruguay; 5 Department of OrthopedicsUniversidad de MontevideoMontevideoUruguay

**Keywords:** distal radius fracture, volar locking plate, dorsal ulnar corner, scaffold fixation

## Abstract

**Objective:**

Volar locking plates (VLPs) are commonly used to treat displaced intra-articular distal radius fractures (DRFs), but multi-fragmented fractures may require additional dorsal fixation. This study assessed the outcomes of a technique combining VLP and a dorsal bone clamp, described here as “scaffold fixation,” and evaluated whether dorsal ulnar corner (DUC) fixation is essential for radiocarpal alignment.

**Methods:**

In this retrospective cross-sectional study of the ICUC database, 87 patients with DRF with preoperative CT-identified DUC fragments were included. The range of motion was measured, and radiocarpal alignment was evaluated on postoperative and final follow-up radiographs. In addition, we compared cases with long screw purchases or short screw purchases in the DUC with respect to DUC size.

**Results:**

No significant differences were found in flexion, extension, supination, or pronation. DUC size was not associated with screw purchase nor with ROM outcomes. Radiocarpal malalignment was seen in 2% of patients postoperatively and 7% at final follow-up, with no difference between screw purchase groups.

**Conclusion:**

Scaffold fixation with a VLP, aided by reduction using a dorsal bone clamp, effectively restores ROM and generally preserves radiocarpal alignment in DRF fixation, regardless of DUC screw purchase or DUC size in this cohort.

**Level of Evidence:**

Level III.

## Introduction

Surgical fixation of comminuted intra-articular distal radius fractures (DRFs) can be challenging. 11 22 Volar locking plates (VLPs) have gained popularity for their ability to provide stable and effective fixation for most DRFs. 33 44 However, several studies have suggested that displaced multi-fragmented intra-articular DRFs cannot be stabilized effectively by volar plating alone and may require additional dorsal and or radial approaches to reduce and capture small but critical articular fragments. 55 66 77 88 99

The most challenging fracture fragments to address in AO/OTA C-type fractures are small fragments of the radial styloid, volar ulnar corner, or dorsal ulnar corner (DUC). 1010 1111 While much has been written about volar ulnar corner escape and the involvement of the radial styloid in radiocarpal fracture dislocation, less has been described regarding the DUC fragment and the ability of a VLP to stabilize it. 1212 1313 1414 1515 The DUC fragment plays a crucial role in maintaining radiocarpal alignment and the stability of the distal radioulnar joint (DRUJ). 1111 1616

To address these challenges, fragment-specific plates or augments to existing VLP have been developed. 77 1717 1818 1919 When only using a VLP, a recent study recommended utilizing longer screws greater than 75% of the volar-radial distal radius width to better capture and stabilize the DUC fragment. 1616 However, screw prominence has its risks, including extensor tendinitis and extensor tendon rupture, leading most surgeons to avoid placing screws with any dorsal cortical penetration. 2020 2121 In addition, one challenge in the existing literature is that most of the recommendations are based on biomechanical studies or small case series of failure, since postoperative computed tomography (CT) scans to assess screw depth, fragment size, and fragment fixation are uncommon. Using pre- and postoperative CT scans, our group previously demonstrated that DUC fractures can often be effectively stabilized with a VLP and bone reduction clamp, creating a so-called “scaffold fixation” construct that relies on subchondral support and interfragmentary compression rather than direct fragment capture. 2222 In this context, scaffold fixation refers to the use of a VLP as a stable volar buttress, combined with controlled reduction of dorsal fragments using a dorsal bone clamp, allowing the locking screws to support the reduced articular fragments indirectly through a subchondral “scaffold” effect. However, that study did not assess the impact of screw length on maintaining reduction or patient outcomes.

**Fig. 1:**
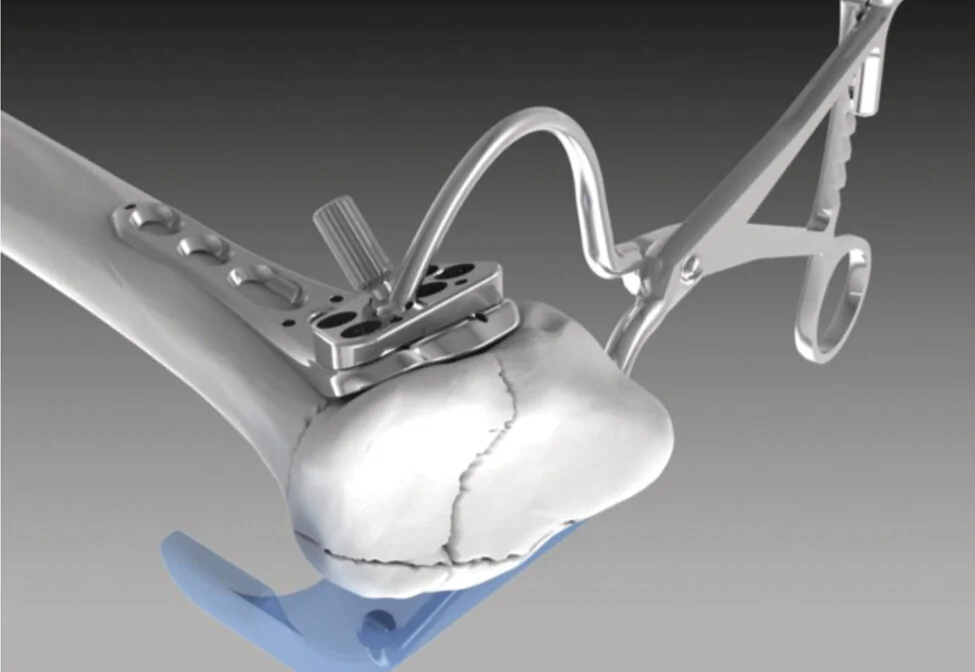
**Fig. 1** Radio lucent bone clamp, applied through the dorsal skin for fracture retention, making use of the VA locking plate (Synthes) as a reduction template.

Analogous to the construction industry, the VLP functions as a “base plate,” while reduction with an adorsal bone clamp allows placement of locking screws close to, but not penetrating, the dorsal ulnar cortex. These screws provide indirect subchondral support, collectively functioning as a scaffold that maintains reduction of the intra-articular fragments ( Fig. 1Fig. 1 ). This biomechanical concept differs from fragment-specific fixation in that stability is achieved through indirect support of the reduced fragments rather than direct fixation of the dorsal-ulnar corner fragment itself. The concept of the dorsal bone clamp for the fixation of DRFs with VLP is further explained in this video: https://www.vumedi.com/video/the-ball-tipped-fracture-reduction-forceps-an-integral-part-of-the-operative-treatment-of-distal-rad/ .

Along with this, in this study, we focused on the DUC to determine if fixation into the DUC fragment is always necessary to maintain radiocarpal alignment when using a VLP to stabilize comminuted intra-articular DRF, and we also assessed the surgical results. We hypothesized that “scaffold fixation” using short screws would not be able to stabilize the DUC fragment in comminuted C-type fractures.

## Materials and Methods

### Study Design

We conducted this retrospective cross-sectional study within the open-access ICUC database, assessing surgical outcome for range of motion (ROM), radiographic radiocarpal alignment, and reported DRUJ instability following treatment of comminuted intra-articular DRF fixation with a VLP and a dorsal bone clamp. This study was Institutional Review Board-exempt as it used publicly available anonymous data.

### Data Set

The ICUC database utilized in this research represents a global collaborative initiative dedicated to the open-access dissemination of data on orthopedic surgical procedures. 2323 The ICUC Database is an anonymized, continuous series of cases recorded at five centers: Freiburg, Luzern, Milano, Montevideo, and Zurich. Continuous and complete registration of each relevant surgical step is collected. This information is kept anonymous and unchanged. For data to be included in the ICUC database, access to follow-up data and informed consent for anonymous public use of the images are mandatory. The database includes for every case: all applicable radiographical imaging (pre- and postreduction, postoperation, and at follow-up), CT scans (postreduction and postoperation), 3D colored models, intraoperative radiographic imaging and photos, and clinical pictures of ROM. All surgeons in the ICUC database are hand-fellowship-trained senior surgeons, defined as highly experienced specialists or experts. 2424

### Patient Variables and Outcomes

**Fig. 2:**
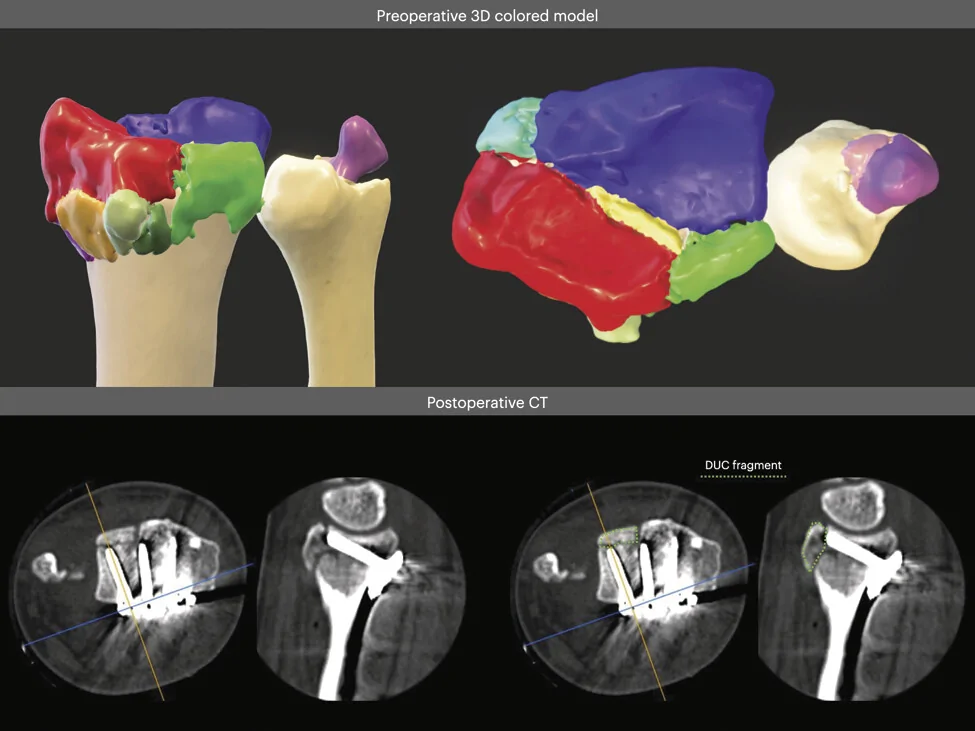
**Fig. 2** Preoperative 3D colored model and postoperative CT to assess dorsal ulnar corner (ICUC ID-654).

We identified all intra-articular comminuted DRF within the database treated with a VLP up to June 2024. We included patients with a dorsal ulnar column fragment on a preoperative CT scan. The presence of the DUC fragment was assessed by two researchers looking at preoperative interactive 3D colored models and immediate postoperative CT for each case ( Fig. 2Fig. 2 ). Exclusion criteria were multiple plate fixation or age < 18 years. The following baseline characteristics were collected: age at the time of injury (years), sex (female/male), time to last follow-up radiograph, and time of final ROM follow-up.

All fractures were treated using a volar angular stable two-column distal radius plate (VA-LCP 2.4, DePuy Synthes, Switzerland).

Injury-specific variables included the degree of comminution with the total number of fragments and the occurrence of concomitant fractures. We classified the DUC fragment size according to feasibility for independent fixation on postoperation CT scanning. Specifically, fragments were categorized as (1) “large” enough for independent fixation, (2) too “small” for independent fixation, or (3) comminuted. Two senior authors independently reviewed the radiographic and 3D reconstruction images and assigned each fragment to one of these three categories. Discrepancies were resolved by consensus. Fixation was also categorized using a binary “long/short” screw purchase variable. Long screw purchase was defined as a screw up to or through the cortex, and short screw purchase was defined as when screws did not reach the dorsal cortex. 1616 1818

The primary outcome included the assessment of the ROM of the affected wrist as assessed through clinical images. ROM assessment was conducted using standardized digital photographs from the ICUC database. Flexion, extension, supination, and pronation were consistently measured by a single researcher (LD) using a digital protractor on these digital photographs to ensure uniformity and minimize interobserver variability. This method provided a standardized approach to evaluating ROM across all patients. The minimal clinical ROM follow-up was 3 months postsurgery. In the cases that had hardware removal ( *n* = 7), the last ROM photo before removal was measured. The occurrence of clinically assessed DRUJ instability was described when reported in the patient reports.

**Fig. 3:**
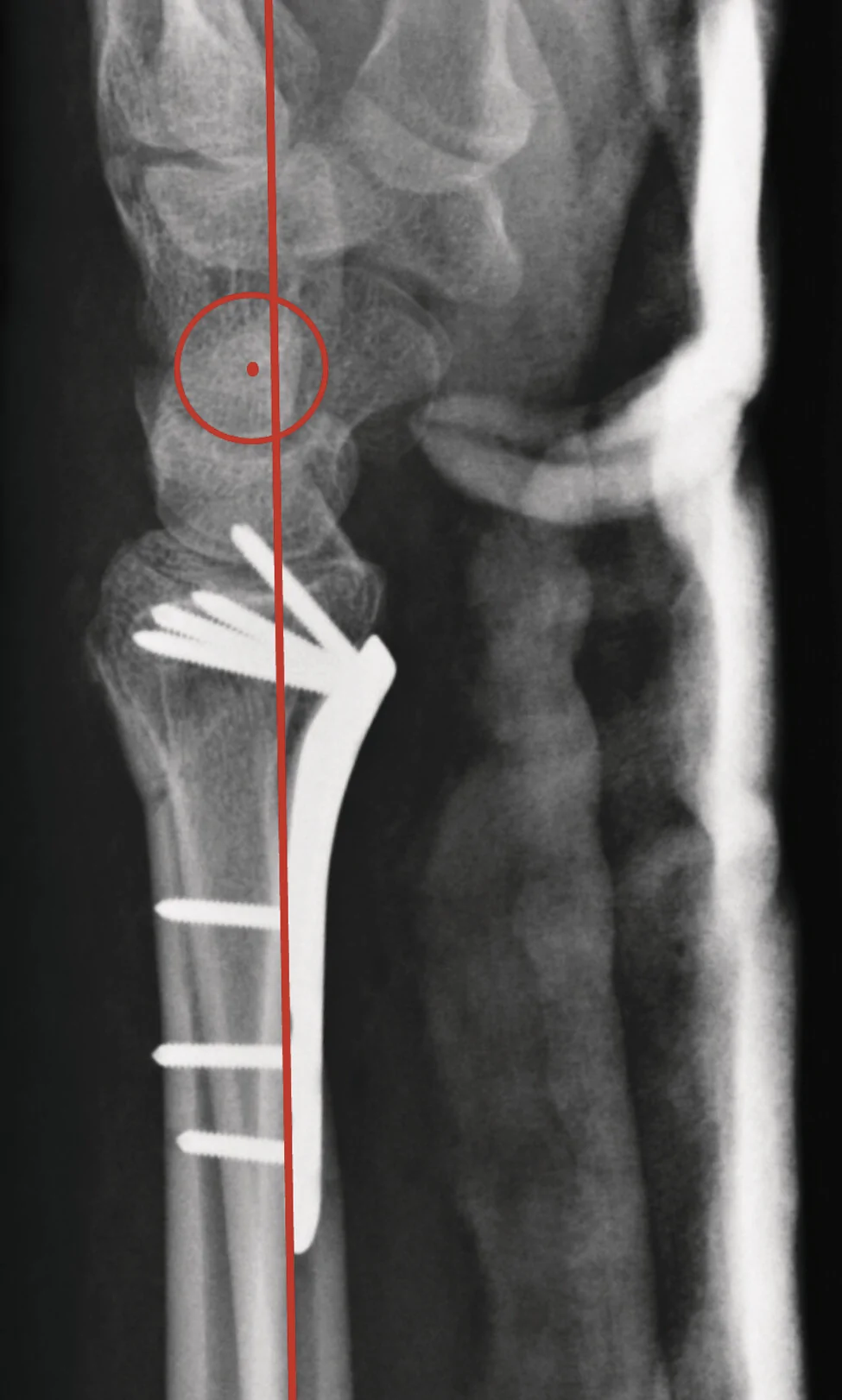
**Fig. 3** Radiocarpal alignment is defined as normal/acceptable when a line drawn from the volar aspect of the distal radius intersects with the capitate head on a lateral radiograph of the wrist (see red line).

The secondary outcome was the achievement of normal radiographic radiocarpal alignment, defined as the alignment of the volar axis of the radius to the capitate head, 2525 2626 as assessed on immediate postoperative radiographs and at final follow-up radiographs, assessed by two independent assessors. The minimal radiological follow-up was 4 weeks. The method used to determine the radiocarpal alignment is shown in Fig. 3Fig. 3 .

### Statistical Analysis

Descriptive statistics were used to summarize patient characteristics, changes in radiocarpal alignment, and final ROM. Data distribution was assessed using the Shapiro–Wilk test. Missing data were not imputed. Continuous data are reported as mean with standard deviation (SD) for normal distributions or median with interquartile range (IQR) for nonnormal distributions. Categorical data are presented as counts with percentages. A two-sided Fisher exact test was used to analyze categorical variables, and an unpaired *t* -test/one-way ANOVA or a Wilcoxon rank sum/Kurskal–Wallis Rank Sum test for continuous variables. To assess the association between screw length and fragment size, a logistic regression analysis was performed. A *p* -value < 0.05 was set to determine statistical significance.

## Results

### Demographics of Cases

**Fig. 4:**
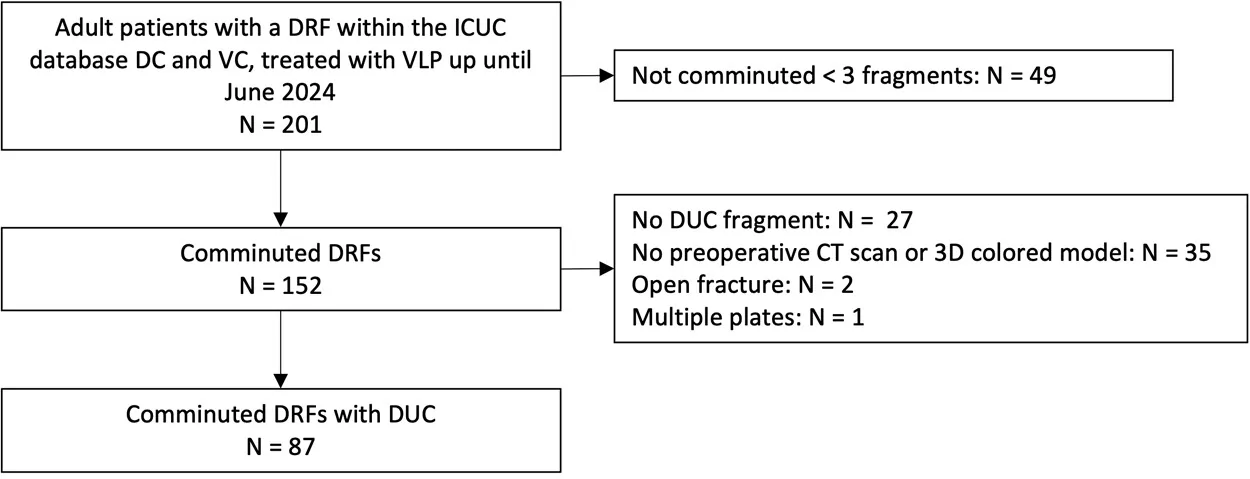
**Fig. 4** Inclusion and exclusion flowchart.

Out of the 123 comminuted DRFs in the ICUC database, we identified 87 DRFs with a DUC fragment treated with a VLP. A flowchart of the inclusion process is shown in Fig. 4Fig. 4 . Baseline characteristics are demonstrated in Table 1Table 1 . Of the included cases, the majority (77%) had four or more fragments. There was a concomitant ulnar styloid fracture in 41 (47%) cases. Short screw purchase was observed in 67 (78%) of the cases. Using small DUC fragments as the reference category, logistic regression showed no association between fragment size and screw purchase length ( Table 2Table 2 ).

**Table 1:** **Table 1** Descriptive statistics of patients and fracture characteristics

Variables	All patients	Screw purchase in DUC		*p* -Value
		Long	Short	
*n*	87	19 (22%)	68 (78%)	
Age at trauma onset, y (IQR)	60 [47.5–70]	65 [60–80]	55 [45–70]	** 0.01 a**
Female, no. (%)	60 (69%)	17 (89%)	43 (63%)	** 0.05 b**
Follow-up radiograph, wk (IQR)	54 [28.5–89]	47 [17.8–87.5]	54.5 [34–89]	0.38 a
Follow-up ROM, wk (IQR)	46 [26–79]	33 [15.5–77.5]	48 [27.5–80]	0.35 a
No. of total fragments (IQR)	4 [4–5]	4 [4–4]	4 [4–5]	** 0.09 b**
Concomitant fractures	41 (47%)	8 (42%)	33 (48%)	0.48 a
Number of fragments				
3	20 (23%)	4 (21%)	16 (24%)	** 0.02 b**
4	37 (43%)	13 (68%)	24 (35%)	
≥5	30 (34%)	2 (11%)	28 (41%)	
Size of DUC fragment				
Small	51 (59%)	10 (53%)	41 (60%)	0.62 b
Large	28 (32%)	8 (42%)	20 (30%)	
Comminuted	8 (9)	1 (5%)	7 (10%)	
Postreduction radiocarpal alignment, no. (%)				
Correct	85 (98%)	18 (95%)	67 (99%)	0.39 b
Incorrect	2 (2%)	1 (5%)	1 (1%)	
Radiocarpal alignment at final follow-up, no. (%)				
Correct	81 (93%)	16 (84%)	64 (84%)	0.15 b
Incorrect	6 (7%)	2 (11%)	4 (6%)	

Note: Value is displayed as median with interquartile range for continuous nonparametric variables, as mean with standard deviation for continuous variables with normal distribution, and as number with percentage for categorical variables.

a Wilcoxon Rank Sum test was done.

b Fisher's exact test was done. Bold indicates significance.

**Table 2:** **Table 2** Logistic regression analysis of DUC fragment size associated with short screw purchase

DUC fragment size	OR	95% CI	*p* -Value
Intercept (small)	4.10	2.14–8.66	<0.01
Large	0.61	0.21–1.82	0.37
Comminuted	1.17	0.26–33.81	0.64

Note: Values in parentheses represent 95% confidence intervals.

### Range of Motion

Ten cases were excluded from analysis due to ROM ≤ 3 months postsurgery, and in one patient, there were no ROM photos available. The median time to final follow-up on ROM was 52.5 weeks (IQR = 26.8–82.3). There was no significant difference between the final ROM in the cases with long screw purchase or short screw purchase concerning flexion, extension, supination, and pronation ( Table 3Table 3 ). Furthermore, there was no significant difference between final ROM and cases with a small, large, or comminuted DUC fragment ( Table 4Table 4 )

**Table 3:** **Table 3** Final range of motion after VLP fixation, assessed for long and short screw purchase

	All patients; *n* = 76 a	Screw purchase in DUC		*p* -Value
		Long; *n* = 14	Short; *n* = 62	
Flexion degree (SD)	70.9 (9.7)	69.3 (6.9)	71.3 (10.3)	0.49 b
Flexion percent of contralateral (SD)	0.90 (0.12)	0.88 (0.11)	0.91 (0.13)	0.47 b
Extension degree (SD)	78.4 (7.5)	75.4 (5.4)	79.1 (7.7)	0.06 b
Extension percent of contralateral (SD)	0.93 (0.08)	0.91 (0.07)	0.94 (0.08)	0.19 b
Supination degree [IQR]	88.5 [83.7–90.2]	88.7 [85.9–90.0]	88.5 [83.2–90.2]	0.87 c
Supination percent of contralateral (SD)	0.99 [0.95–1.00]	1.00 [0.98–1.02]	0.98 [0.94–1.00]	0.13 c
Pronation degree [IQR]	89.3 [85.1–90.6]	90.2 [87.9–92.0]	89.2 [85.1–90.5]	0.27 c
Pronation percent of contralateral (SD)	1.00 [0.97–1.02]	1.00 [0.97–1.02]	0.99 [0.96–1.02]	0.94 c

Note: Value is displayed as mean with standard deviation for continuous variables with normal distribution and as median with interquartile range for continuous nonparametric variables, as mean with standard deviation for continuous variables with normal distribution, and as number with percentage for categorical variables.

a Additional *n* = 3 were excluded from the ROM percent of contralateral due to bilateral wrist fractures.

b Unpaired *t* -test was done.

c Wilcoxon rank sum test was done. *n* = 11 were excluded from analysis due to ROM not assessed, or ≤ 3 months postsurgery.

**Table 4:** **Table 4** Final range of motion after VLP fixation, assessed DUC fragment size

	All patients; *n* = 76	DUC fragment size			*p* -Value
		Small; *n* = 44	Large; *n* = 21	Comminuted; *n* = 8	
Flexion degree (SD)	70.9 (9.7)	71.9 (10.9)	69.6 (6.8)	69.7 (9.6)	0.61 a
Flexion percent of contralateral (SD)	0.90 (0.12)	0.91 (0.14)	0.88 (0.12)	0.88 (0.09)	0.57 a
Extension degree (SD)	78.4 (7.5)	79.7 (7.9)	76.5 (6.1)	76.4 (7.7)	0.19 a
Extension percent of contralateral (SD)	0.93 (0.08)	0.94 (0.08)	0.93 (0.06)	0.92 (0.07)	0.59 a
Supination degree [IQR]	88.5 [83.7–90.2]	88.9 [84.0–90.4]	88.5 [86.3–90.2]	84.3 [82.0–87.4]	0.20 b
Supination percent of contralateral (SD)	0.99 [0.95–1.00]	0.99 [0.96–1.01]	0.99 [0.97–1.02]	0.95 [0.93–0.99]	0.46 b
Pronation degree [IQR]	89.3 [85.1–90.6]	89.4 [85.0–90.8]	89.2 [86.9–90.5]	87.0 [74.2–90.9]	0.65 b
Pronation percent of contralateral (SD)	1.00 [0.97–1.02]	1.01 [0.98–1.02]	0.99 [0.96–1.01]	0.96 [0.92–1.00]	0.06 b

Note: Value is displayed as mean with standard deviation for continuous variables with normal distribution and as median with interquartile range for continuous nonparametric variables, as mean with standard deviation for continuous variables with normal distribution, and as number with percentage for categorical variables.

a One-way ANOVA was done.

b Kurskal–Wallis rank sum test was done. Bold indicates significance. *n* = 11 were excluded from analysis due to ROM not assessed, or ≤ 3 months postsurgery. An additional *n* = 3 were excluded from the ROM percent of contralateral due to bilateral wrist fractures.

Compared with a contralateral uninjured wrist, flexion, extension, supination, and pronation percentages did not significantly differ between groups ( Table 3Table 3 ). Furthermore, there was no significant difference between the final ROM compared with a contralateral uninjured wrist and cases with a small, large, or comminuted DUC fragment ( Table 4Table 4 )

### Radiocarpal Alignment

On immediate postoperative radiographs, the radiocarpal alignment was abnormal in two cases (2% of the total 87 cases). Of which, one case had a long screw purchase and one case had a short screw purchase, with no significant difference between the groups (5% vs. 1%, *p* = 0.39). Both cases had a small DUC fragment.

At final follow-up radiographs, four additional cases showed loss of radiocarpal alignment, resulting in a total of six (7%) cases: two (11%) cases had long screw purchase and four (6%) cases had short screw purchase. There was no significant difference in radiocarpal alignment at follow-up radiographs between cases with long and short screw purchases ( *p* = 0.15). Half of the cases had a small DUC fragment, and half had a large one. Finally, in four cases, there was clinically reported DRUJ instability, three having short screw purchase and one long screw purchase. Revision surgery was not performed.

## Discussion

Scaffold fixation by inserting a VLP over fracture fragments reduced with a dorsal bone clamp represents a treatment strategy for stabilizing a multi-fragmented intra-articular DRF that avoids independent DUC fixation. In this technique, the dorsal clamp plays a pivotal role by allowing controlled and stable reduction of the dorsal fragments, particularly the DUC, prior to volar plating. This could potentially minimize the need for separate dorsal hardware and may reduce soft-tissue disruption. In this retrospective cohort study, we observed that the final ROM of patients with either long or short screw purchase in the DUC did not differ in ROM outcomes. Similarly, DUC fragment size was not associated with differences in ROM outcomes. Furthermore, we observed preserved radial carpal alignment, with no significant difference between short and long screw purchase in the DUC within this cohort, supporting the stabilizing contribution of the dorsal reduction clamp in achieving and maintaining anatomical alignment.

### Strengths and Limitations

This study should be interpreted considering its strengths and limitations. This study describes outcomes of a cohort treated with a scaffold fixation technique using a VLP, providing insight into this approach for DRFs involving a dorsal-ulnar corner fragment.

It uses a scaffold technique in securing the surrounding fragments and considers that soft tissues are essential to achieve fracture reduction and increase construct strength. In addition, this study included an openly accessible database at ICUC.net with intraoperative imaging and follow-up information.

A key limitation of this study is the absence of a comparison group. Without a control cohort treated with alternative fixation strategies, such as dorsal plating or fragment-specific fixation, no causal inferences can be made regarding the effectiveness or equivalence of scaffold fixation. The findings should therefore be interpreted as descriptive outcomes of a cohort treated with this technique rather than evidence of superiority or comparative effectiveness. Future research should include comparative studies with appropriate control groups to determine whether scaffold fixation provides clinically or radiographically meaningful benefits over established fixation strategies, particularly with respect to functional outcomes, radiocarpal alignment, and joint stability.

Second, the measurement of radial carpal alignment was retrospectively assessed by one observer on radiographs. The exact position of the wrist on the lateral radiographs could have varied between cases. In addition, the radial carpal alignment can be measured in multiple ways. In this study, we have chosen the method to determine the radial alignment by radiocarpal distance in line with the width of the capitate, showing excellent intra- and inter-observer agreement. 2525 2626 However, the exact measurement of the capitate-to-axis-of-radius distance was not assessed.

Third, the DRUJ assessment is essential for assessing the wrist's stability—especially in the dorsal-ulnar corner. However, there is no consensus in the research on whether this can be accurately measured using an imaging modality. 2727 2828 Clinical assessment of DRUJ stability is the most reliable method, but it could not be assessed in the study due to its retrospective design. A prospective study with long-term follow-up and serial assessment of DRUJ stability could better assess another aspect of capturing the DUC with a screw.

Lastly, complication-related outcomes were not evaluated in this study. Although complications such as extensor tendon disorders, infection, nonunion, hardware-related problems, and reoperations are clinically relevant, these events are currently not consistently or systematically registered within the openly accessible ICUC.net database. As a result, reliable extraction and analysis of complication data were not feasible for the present cohort. Future prospective studies with standardized and dedicated complication reporting are required to more comprehensively assess the safety profile of scaffold fixation.

In addition, this study only included patients with a dorsal-ulnar corner fragment. Future research must include broader cases with different specific fracture fragment patterns to assess whether the scaffold fixation method is accurate and generalizable.

### Final ROM

Our study demonstrated adequate restoration of ROM in absolute terms and in relation to the contralateral uninjured wrist motion. Previous literature on the fixation of DRFs with dorsal-ulnar corner fragments has shown comparable or less favorable results concerning both absolute ROM and percentage of the contralateral. 2929 3030 3131 ROM is a crucial clinical outcome from the patient's perspective. This study indicates that the final ROM is not adversely affected when either long or short screw purchase in the dorsal-ulnar corner fragment is used. The results showed comparable outcomes with the nonfractured wrist, suggesting that functional wrist motion can be achieved using this technique in the studied cohort.

### Radiocarpal Alignment

When fixating a DRF with a dorsal-ulnar corner fragment, it is usually advised to fixate the dorsal-ulnar corner fragment with a long locking screw placed through the VLP, dorsal plate, recontoured plate, fragment-specific fixation, or other adjunct methods. 3232 3333 The scaffold approach proposed in this study is a new technique that does not focus on the fragment-specific approach advised for comminuted DRFs. In our cohort, radiocarpal alignment was generally preserved; however, loss of alignment was observed in a small subset of cases at final follow-up. Our study did not demonstrate an association between screw purchase length in the dorsal-ulnar corner and radiocarpal alignment. Due to the limited number of maligned cases, further statistical analysis to identify predictors of loss of alignment was not feasible. Shorter screws that are less likely to cause extensor tendon irritation may be considered with the expectation of similar ROM and maintenance of distal radial alignment. Further research should include comparative studies with an appropriate control group to evaluate the potential clinical and radiographic benefits of this approach. One study similarly demonstrated that the majority of DRFs fixated by a single VLP achieve union without loss of reduction. 3434 Additionally, another study indicated that the size of the dorsal-ulnar corner fragment does not indicate the need for a separate dorsal approach in the fixation of a DRF, which also aligns with our findings. 3535 Furthermore, a study reported that the stability of comminuted DRFs with volar fragmentation is determined by the reduction of the major fragments and the reduction of the small volar lunate fragment or dorsal-ulnar corner. 1212 However, this conclusion was a case series based on seven patients with volar shearing fractures. In Beck et al, 3131 52 type B3 DRFs involving the lunate facet were treated with standard VLP, and the incidence of loss of reduction was 13%. One additional study on type B3 DRFs did not show any reduction in the loss of fractures after VLP. 3030 Souer et al 2929 conducted a multicenter review of 57 volar marginal rim fractures treated with standard volar plating and noted a 4% reduction in the loss rate at the final follow-up.

### Plating

When treating DRF, using a single VLP with a dorsal bone clamp offers several advantages over multiple plating techniques, particularly in terms of surgical ease, early mobility, and reduced soft tissue complications. 3636 3737 3838 The volar approach is less invasive, minimizing the risk of soft tissue irritation and injury, a common issue with dorsally inserted plates. 2020 2121 3939 This method simplifies the surgical procedure and promotes faster recovery and rehabilitation. While dorsal locking plates provide direct fracture exposure and benefit specific fracture types like dorsal shear fractures and dorsal die-punch fractures, they come with increased risks of soft tissue problems. 4040 4141

In conclusion, this study reports that scaffold fixation was associated with good functional ROM outcomes and generally preserves radiocarpal alignment in a cohort of patients with multi-fragment intra-articular DRFs, regardless of DUC fragment size. Given the absence of a comparison group, these findings should be interpreted as descriptive rather than evidence of superiority over other fixation methods. Furthermore, screw length did not contribute to the maintenance of DUC stability, especially radiocarpal alignment and DRUJ stability, suggesting that scaffold fixation with VLP, dorsal bone clamp, and shorter screws may be a viable option without compromising radiographic outcomes or ROM in key movement arcs. Future research is needed to evaluate the long-term effects of scaffold fixation on joint stability, particularly regarding DRUJ stability, patient-reported outcomes, and functional recovery.
